# Hydroponic Solutions for Soilless Production Systems: Issues and Opportunities in a Smart Agriculture Perspective

**DOI:** 10.3389/fpls.2019.00923

**Published:** 2019-07-24

**Authors:** Paolo Sambo, Carlo Nicoletto, Andrea Giro, Youry Pii, Fabio Valentinuzzi, Tanja Mimmo, Paolo Lugli, Guido Orzes, Fabrizio Mazzetto, Stefania Astolfi, Roberto Terzano, Stefano Cesco

**Affiliations:** ^1^ Department of Agronomy, Food, Natural Resources, Animals and Environment (DAFNAE), University of Padova, Legnaro, Italy; ^2^ Faculty of Science and Technology, Free University of Bozen-Bolzano, Bolzano, Italy; ^3^ Department of Agricultural and Forestry Sciences (DAFNE), University of Tuscia, Viterbo, Italy; ^4^ Department of Soil, Plant and Food Sciences, University of Bari, Bari, Italy

**Keywords:** nutrient acquisition, biofortification, nutrient interaction, plant growth-promoting rhizobacteria, nanoparticles, sensors, smart agriculture

## Abstract

Soilless cultivation represent a valid opportunity for the agricultural production sector, especially in areas characterized by severe soil degradation and limited water availability. Furthermore, this agronomic practice embodies a favorable response toward an environment-friendly agriculture and a promising tool in the vision of a general challenge in terms of food security. This review aims therefore at unraveling limitations and opportunities of hydroponic solutions used in soilless cropping systems focusing on the plant mineral nutrition process. In particular, this review provides information (1) on the processes and mechanisms occurring in the hydroponic solutions that ensure an adequate nutrient concentration and thus an optimal nutrient acquisition without leading to nutritional disorders influencing ultimately also crop quality (e.g., solubilization/precipitation of nutrients/elements in the hydroponic solution, substrate specificity in the nutrient uptake process, nutrient competition/antagonism and interactions among nutrients); (2) on new emerging technologies that might improve the management of soilless cropping systems such as the use of nanoparticles and beneficial microorganism like plant growth-promoting rhizobacteria (PGPRs); (3) on tools (multi-element sensors and interpretation algorithms based on machine learning logics to analyze such data) that might be exploited in a smart agriculture approach to monitor the availability of nutrients/elements in the hydroponic solution and to modify its composition in *realtime*. These aspects are discussed considering what has been recently demonstrated at the scientific level and applied in the industrial context.

## Introduction

Considering that human world population will reach about 9 billion by the year 2050 (Tilman et al., 2002), it appears clear that food security is one of the pivotal themes of the new millennium and, reasonably, the most urgent challenge for the agricultural sector. However, it should be considered that the progressive drop of fertile soil surface, due to environmental pollution and urbanization phenomena (Chen, 2007), greatly complicates the context. In this regard, the intensification of the production cycles and the monoculture approach, which favored the diffusion of many pathogens and the development of the corresponding pathologies (Jones et al., 1991; Shafique et al., 2016; Tittarelli et al., 2016), should also be taken into account. Moreover, the strict dependency of agricultural practice on water availability (Verones et al., 2017) in an age of drastic climate changes (desertification) makes the scenario even more complex. In this respect, the possibility of exploiting surfaces not anymore fertile (due to pollution or pathogen problems) for agricultural purposes and also limiting at the same time the water consumption (see also Table 1; Pignata et al., 2017; Müller et al., 2017) makes the soilless system cultivation surely a valid opportunity. Moreover, it should be highlighted that this cultivation approach also represents a favorable response toward a more environmentally friendly agriculture (Benke and Tomkins, 2017; Maucieri et al., 2017; Tajudeen and Taiwo, 2018) as well as a promising tool also in the vision of the general challenge of food security.

**Table 1:** Water use efficiency (WUE) in soilless and soil cultivation systems of several crops.

Type of efficiency	Species	Crop water requirement (L kg^−1^)		
		Soilless cultivation system		Soil cultivation system*
WUE	Lettuce	1.6	Barbosa et al., 2015	76
	Hot pepper	58	Ahmed et al., 2014	110
	Sweet pepper	17	El-Sayed et al., 2015	121
	Zucchini Squash	39	Rouphael et al., 2005	97
	Muskmelon	42	Hamdy et al., 2002	170
	Spinach	8.3	Van Ginkel et al., 2017	106
	Strawberries	136	Van Ginkel et al., 2017	544
	Brassica	5.0	Van Ginkel et al., 2017	129
	Tomatoes	35	Massa et al., 2010	78

* *Values were estimated using FAO references (http://www.fao.org/3/u3160e/u3160e04.htm) adopting a sub-humid cultivation area and an average ETo of 5 mm day^−1^. The following formula was used to calculate the crop evapotranspiration: ETc = [(kc × ETo) × average length of total growing season (days)] where ETo is the reference crop evapotranspiration and kc is the crop factor. kc values were used according to Dukes et al. (2012)*.

Currently, about 3.5% of the worldwide area cultivated under tunnels and greenhouses for vegetables production adopts the soilless agriculture techniques based on hydroponic solution (such as floating systems, nutrient film technique (named also NFT) – or aeroponics, Hickman (2016)). This significant diffusion at the field scale undoubtedly highlights the presence of many advantages of this production approach in addition to the more efficient use of the nutritional resources including water (Kinoshita et al., 2016; Rodriguez-Ortega et al., 2017). In fact, there is a variety of examples where hydroponic solution can be efficiently used for biofortification programs with oligo elements (e.g., iodine (I), selenium (Se), silicon (Si), and calcium (Ca), see also Table 2; Schiavon et al., 2013; Tomasi et al., 2013, 2015a,b; Smoleń et al., 2016; D’Imperio et al., 2016a,b; Mimmo et al., 2017) as well as to improve vegetable quality and its shelf life according to the market and consumer needs (Giuffrida et al., 2014, 2017; Amalfitano et al., 2017; Giro and Ferrante, 2017; Yasuor et al., 2017; Islam et al., 2018). However, the extensive research activity aimed at fine-tuning fertilizers’/nutrients’ concentration in the hydroponic solutions, particularly (1) to restrain nitrate content in edible plant tissues (Fageria, 2010), (2) to guarantee vegetables’ safety, and (3) to improve the nutritional quality of the yields (Fallovo et al., 2009; Gruda, 2009; Putra and Yuliando, 2015), point out that there is still much room for improvement. This is particularly true in the light of the recent scientific pieces of evidence about the mechanisms underlying the mineral nutrition in plants and their regulation (competition/antagonism/interaction among nutrients) and about the bio-geochemical cycles of nutrients in the soil solution (solubilization/precipitation). This knowledge is fundamental when the development of different strategies (including approaches and tools such as beneficial microorganisms-PGPRs and nanoparticles) for the optimization of the hydroponic production of vegetables is pursued.

**Table 2:** Mineral biofortification in soilless vegetables species (research activities are represented by coloured cells).

Species	I	Se	Si	Ca	References
*Brassica spp.*					Weng et al., 2008; Ávila et al., 2014; D’Imperio et al., 2016a,b; Wiesner-Reinhold et al., 2017
*Capsicum annum*					Li et al., 2017
*Cichorium inthybus*					D’Imperio et al., 2016a,b
*Fragaria* × *ananassa*					Mimmo et al., 2017
*Lactuca sativa*					Park et al., 2009; Garibaldi et al., 2012; Smoleń et al., 2014
*Solanum lycopersicum*					Stamatakis et al., 2003; Nancy and Arulselvi, 2014;
*Spinacia oleracea*					Zhu et al., 2003; Ferrarese et al., 2012; D’Imperio et al., 2016a
*Valeraniella locusta*					Gottardi et al., 2012

In the industrial context, a new paradigm labeled as Industry 4.0 or Smart Manufacturing and based on cyber-physical manufacturing systems and Internet of Things is emerging (Kagermann et al., 2013). It is interesting to note that hydroponic production of vegetables carried out in limited and well-controlled environments is going to reduce the most relevant differences between agricultural and industrial processes and, in turn, to improve the quality control. However, to achieve this goal, a set of additional monitoring tools specifically for the hydroponic-based cultivation approach are necessary. In this regard, sensors for *realtime* monitoring of the hydroponic solutions composition (i.e., availability of nutrients/elements) as well as interpretation algorithms also based on machine learning logics to analyze such data play a pivotal role. In fact, only thanks to these tools, that can be borrowed from the industrial context, it might be possible to maintain/adapt in *realtime* the composition of a hydroponic solution in order to achieve products of a desired quality.

This review is aimed at analyzing the open questions of hydroponic systems and at highlighting opportunities in their applicative use in a field scale, considering also what has recently been demonstrated at the scientific level and applied in industrial context. In particular, from the scientific point of view, we would like to unravel the topics and problems that have been adequately studied and the ones that instead still require significant research efforts, all these pieces of information being essential for a better management of crop nutrient acquisition in soilless systems. Moreover, from the practical point of view, the potential to use new forms of nutrients and/or bioeffectors as well as new technologies to collect/analyze data could be a viable tool at the field scale for farmers in a context of a smart agriculture.

## Open Issues of Hydroponic Solutions Used in Soilless Agriculture Techniques

It is widely known that the productivity and quality of crops grown in hydroponic systems are markedly dependent on the extent of the plant nutrients acquisition from the growing medium (Valentinuzzi et al., 2015). It is interesting to highlight that this root physiological process is not only affected by the availability levels of the nutrients (i.e., by their soluble forms) in the medium (solubilization/precipitation: section “Chemical Management of Nutrient Availability in the Hydroponic Solution”), but also by the nutrient sources (nutrient chemical forms: section “Nutrient Chemical Forms and Uptake Processes”) and/or by the interactions among the different nutrients (e.g., competition/antagonism: section “Nutrient Interactions”).

### Chemical Management of Nutrient Availability in the Hydroponic Solution

When dealing with hydroponic cultures, solution chemistry is fundamental to ensure adequate nutrient concentrations for plant uptake. In particular, multiple chemical equilibria must be taken into account when preparing nutrient solutions using salts or concentrated liquid stocks, especially solubilization/precipitation equilibria (De Rijck and Schrevens, 1998b). In fact, a number of physical-chemical phenomena can alter the nutrient availability for plants, the most important of which are (1) precipitation, (2) co-precipitation, and (3) complexation. In this respect, it should be highlighted that the temperature of the nutrient solution, affecting the chemical equilibria in solution, may considerably influence these processes. This is particularly crucial for areas where the overwarming of the nutrient solution often occurs, impacting also at the physiological level of crops (Lee and Takakura, 1995 – spinach; Fazlil Ilahi et al., 2017 – butterhead lettuce).

Precipitation reactions may occur when cations and anions in aqueous solution combine to form an insoluble ionic solid (the precipitate). Such conditions, called saturation, occur when the concentrations of certain cations and anions in solution reach a maximum limit value (solubility). The concentrations of ions in equilibrium with the precipitate (i.e., solubility) can be calculated using a specific equilibrium constant called solubility product, which is tabulated for many chemical compounds and depends on temperature. Besides temperature, precipitation equilibria can be also influenced by other parameters such as pH and ionic strength (a parameter that considers the sum of the concentrations of all the ionic compounds in solution and their charge). Cations may form insoluble hydroxides at alkaline pH (by combining with OH^−^ anions) or other insoluble precipitates by reacting with other anionic nutrients; thus, they must be carefully balanced and optimized to avoid losses from solution. In such cases, the values of pH and those of redox potential (Eh) must be continuously monitored or controlled. In this regard, pH values above 7 and positive Eh values may cause the precipitation of nutrients like iron (Fe), zinc (Zn), copper (Cu), nickel (Ni), and manganese (Mn^II^) as insoluble (hydr)oxides. Precipitation of Fe^III^ may occur already at pH well below the neutrality (Takeno, 2005). At negative Eh values and acidic pH, e.g., in uncontrolled hydroponic systems under anoxic conditions, the same elements might also precipitate as insoluble sulfides, when sulfate is reduced to sulfide. At high pH values and high dissolved CO_2_ concentrations, macronutrients like Ca and magnesium (Mg) can precipitate as carbonates. Precipitation of phosphates (mostly hydrogen phosphates) is another process to avoid in hydroponic solutions. This process, besides depleting phosphorus (P) from nutrient solution, may also reduce the solubility of other nutrients such as Ca, Mg, Fe, and Mn^II^. It is known that phosphate availability can be reduced at pH above 7 mostly due to precipitation with Ca. Different Ca-phosphate minerals can potentially form above this pH such as hydroxylapatite [Ca_5_(PO_4_)_3_OH], amorphous tricalcium phosphate [Ca_3_(PO_4_)_2_], and Ca_4_H(PO_4_)_3_·3H_2_O (Lee et al., 2017). Also sulfur (S) availability can be limited by precipitation with Ca, as Ca-sulfate minerals (Packter, 1974). Silicon solubility is usually reduced at acidic pH, where SiO_2_ precipitates may be produced (Takeno, 2005). Precipitation/dissolution phenomena are often promoted by pH changes and therefore pH must be continuously controlled or buffered. Addition of nutrients in the form of salts to hydroponic solutions may lead to hydrolysis reactions, which may result in the acidification or alkalinization of the medium. Nitrogen (N) supply may also alter solution pH, if N is added only in the form of NO_3_^−^ (alkalinization) or NH_4_^+^ (acidification) (Asher and Edwards, 1983). Yet, both N forms are usually added to hydroponic solutions. In general, saturation conditions for a certain nutrient could be reached if its concentration is increased due to water evaporation from the hydroponic system (owing to high temperatures or plant evapotranspiration). However, it has been recently observed that water losses by 20% (or even more) do not significantly influence precipitation equilibria (Tomasi et al., 2015a).

Strictly connected with precipitation are co-precipitation phenomena. These latter refer to the entrapment of an element (usually trace metals) within the structure of an insoluble compound (normally not containing that element) during its precipitation from solution. Co-precipitation may strongly reduce the solubility of nutrients added at trace concentrations like Cu, Zn, Mn^II^, and Ni, when insoluble species like Fe (hydr)oxides, Ca-carbonates, or Ca-phosphates are formed (McBride, 1994). Co-precipitation is a process capable of strongly reducing the solubility of an element, well below that of the least soluble pure mineral phases of the element likely to form under environmental conditions (Martínez and McBride, 2000).

Another important process to consider in hydroponic solutions is complexation, i.e., the formation of a chemical compound where a metal nutrient is bound by one or more neutral molecules or anions (ligands), either of organic or inorganic nature. The resulting complex can be a neutral compound, a cation, or an anion, depending on whether positive or negative charges prevail. Complexation reactions diminish the concentration of the free ions in the nutrient solution, changing elemental bioavailability. In general, the formation of complexes increases metal solubility even if very often, for some nutrients, they are less available for plant uptake than their free ions (De Rijck and Schrevens, 1998a). The addition, organic ligands such as EDTA, DTPA, EDDHA, citrate, etc. can increase the stability of certain elements in solution, especially Fe, Cu, Zn (Lucena, 2003). However, as discussed later in this review, the different complexes may differently affect plant nutrient uptake and allocation (e.g., the case of Fe acquisition). In addition, some forms of soluble hydrogen carbonates and phosphates, as well as chlorides, can reduce the concentration of actual free metal cations in solution through complexation. The stability of complexes is another parameter that must be taken into account when preparing a nutrient solution: while on one side, efficient complexing agents can facilitate the solubilization of a nutrient in water, on the other side, strong complexes are usually more difficultly usable by plants (Kraemer et al., 2006).

Considering all these aspects, it seems thus likely that the composition of a real hydroponic solution could reasonably be different from the planned one; being an altered availability of the nutrients in the growing medium a known risk for the quality of the vegetables (Tomasi et al., 2015a), the availability of tools for a *realtime* analysis of the nutrient solution composition is undoubtedly of particular usefulness and interest.

For a more accurate calculation of chemical equilibria in solution, ion activity should be considered instead of concentration. In fact, ion concentrations can be used only in calculations regarding ideal solutions, i.e., diluted solutions where no interactions among solutes occur. In real solutions, like in hydroponic solutions, interactions among solutes cannot be neglected and therefore ion activity should be used in calculations instead of concentrations. An ion activity is determined by multiplying the concentration of that ion in solution for an activity coefficient (≤1), which depends also on the concentration of all other ions in solution and their charge, i.e., on the ionic strength of the solution. Ion *active* concentration diminishes as the concentration of electrolytes in solution increases. For highly diluted (ideal) solutions, the activity coefficient for the chemical species in solution can be approximated to 1, and therefore ion concentrations are not influenced by phenomena such as ion-pair formation or ion conductivity reduction. But, when dealing with more concentrated solutions of strong electrolytes, like in hydroponic solutions, these phenomena become relevant and may reduce the ion *active* concentration. For example, for a sole KNO_3_ solution at 6 mM concentration (as normally present in Hoagland nutrient solution), the activity coefficient for KNO_3_ is about 0.92 and therefore its *active* concentration is 8% lower than the concentration actually added to solution. However, the activity coefficient of a single electrolyte depends on the ion strength of the whole solution and decreases with increasing concentration of all the electrolytes dissolved and of their charge. Usually, the concentrations of electrolytes in nutrient solutions for soilless cultivation are quite high, in the range 6.4–37.8 meq L^−1^ (corresponding to EC in the range 0.8–4.0 dS m^−1^) (Savvas, 2003), and therefore the ion activity can be significantly reduced (e.g., the activity coefficient for KNO_3_ is about 0.85, with an overall reduction of 15% active concentration).

All the abovementioned processes can be simulated and foreseen by using dedicated software to manage chemical equilibria calculations. Among these, Visual MINTEQ (Gustafsson, 2013), MINEQL+ (Environmental Research Software, USA), CHEMEQL (Müller, 2015), CHEAQS (Verweij, 2017) are some of the most used both in water and soil chemistry. All the mathematical models employed by these software tools are based on thermodynamic data and usually kinetic parameters are not taken into account. However, it is known that kinetic constraints can prevent a process from occurring or limit its representativeness (Terzano et al., 2015). For instance, with respect to the precipitation processes, oversaturation or under-saturation phenomena can change nutrient solubility, respectively, above or below the concentration allowed by thermodynamics.

In a context of a more sustainable agriculture, a specific comment must be dedicated to the exhausted hydroponic solutions at the end of a productive cycle. In this regard, it is clear that they represent an interesting resource in terms of water and fertilizer savings, which is becoming increasingly relevant especially in those countries where there is shortage of rain or good-quality water and farmers cannot afford the expenses to buy large amounts of fertilizers. Moreover, recycling exhausted solutions may also represent an efficient strategy to prevent groundwater and environmental pollution, especially from intensive agricultural productions. However, the main problem with the reuse of exhausted nutrient solutions is the shortage of some key macro and micronutrients (da Silva Cuba Carvalho et al., 2018) and their increased salinity (Grattan and Grieve, 1998; Pardossi et al., 2005; Bar-Yosef, 2008) causing, in turn, problems for crops (Carmassi et al., 2005; Parida and Das, 2005) even if to different extents from one plant species to another (Bar-Yosef, 2008). Thus, a research challenge in this context is surely to develop management practices/tools that reduce salinity in recycled solutions and/or minimize the physiological impact of salinity on plants (Neocleous et al., 2017). In addition, considering the composition of a typical Hoagland solution and according to chemical equilibrium models (Visual Minteq 2.61), at pH 5.5, according to thermodynamic data, few other nutrients could easily reach oversaturating conditions if certain ions accumulate in exhaust Hoagland solutions, mostly phosphates (precipitating as Ca, Mg, or Fe phosphates) and molybdate (as Ca molybdate). Also, this aspect needs a careful monitoring when a reuse of the hydroponic solution for another cycle is planned.

**Figure 1:**
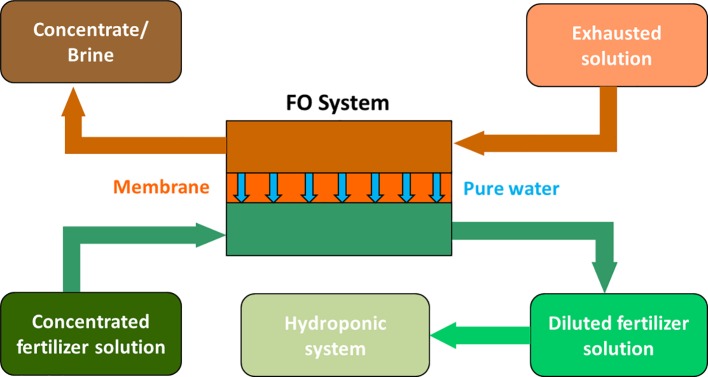
Scheme of a fertilizer drawn forward osmosis desalination process for nutrient solution preparation to be used in hydroponic cultivation systems (modified from Phuntsho et al., 2012).

Salinity increase could be contrasted by treating the recycled water with appropriate osmotic systems, including forward and reverse osmosis. In the last years, particular attention has been paid to forward osmosis (FO) technologies, also with the purpose to reuse wastewater for fertigation purposes (Van der Bruggen and Luis, 2015). In this sense, Phuntsho et al. (2012) proposed the use of concentrated fertilizer solutions as draw solutions in FO systems. This application maybe of interest for hydroponic cultures particularly when the feed water is of a low quality, like an exhausted nutrient solution or wastewater. The concentrated fertilizer solution is used to withdraw pure water from this source (see the scheme in Figure 1). In this way, water is recycled and is also enriched in those nutrients that have been lost by plant uptake or other chemical processes (e.g., precipitation, complexation, sorption). This technology, called fertilizer drawn forward osmosis (FDFO), has been recently applied to grow hydroponic lettuce using a commercial nutrient solution (Chekli et al., 2017). In a pilot-scale study, these authors demonstrated that the FDFO process is able to produce the required nutrient concentration and final water quality (i.e., pH and conductivity) suitable for hydroponic applications and the hydroponic lettuce showed similar growth patterns as the control without any sign of nutrient deficiency.

Another relevant issue when dealing with closed-loop hydroponic cropping systems is the accumulation of potentially toxic organic compounds released by the roots of cultivated plants. This phenomenon is known as allelopathy/autotoxicity (Kitazawa et al., 2005; Asaduzzaman and Asao, 2012) and occurs when a plant species releases chemical substances which inhibit or delay germination and/or growth of the same plant species (Singh et al., 1999; Mondal et al., 2015). Among these substances, benzoic acids were mainly found to inhibit the growth and yield of several crops (Hosseinzadeh et al., 2019). To overcome this issue, a number of treatment techniques have been proposed for root exudates degradation or removal, either physical (e.g., activated carbon adsorption, ion exchange resins, membrane filtration, sand filtration); chemical (e.g., TiO_2_ photocatalysis, UV treatments, ozonation, electrodegradation); or biological (e.g., use of degrading microbial strains) (Asaduzzaman et al., 2012; Mondal et al., 2013; Hosseinzadeh et al., 2017; Talukder et al., 2018, 2019). All these treatments may have advantages and drawbacks as reviewed by Hosseinzadeh et al. (2017). In general, to be effective, the treatment technique applied should be able to remove root exudates without interfering with the inorganic mineral nutrients in solution. One of the main problems in this sense is the degradation/adsorption of Fe chelates (Ehret et al., 2001).

### Nutrient Chemical Forms and Uptake Processes

With respect to the chemical forms of a nutrient, N represents one of the most explicative examples. In fact, it is well demonstrated that plants can use a wide variety of N forms, ranging from the inorganic, namely NH_4_^+^ and NO_3_^−^, to the organic ones, as for instance urea and amino acids (Nacry et al., 2013). Due to environmental conditions and plant species, the relative contribution of each N form to plant N supply can be quite variable (Haynes and Goh, 1978; Schimel and Chapin, 1996; Marschner, 2012). From a metabolic point of view, the assimilation of NH_4_^+^ is less expensive as compared to NO_3_^−^, NH_4_^+^ being directly usable to generate glutamine (Marschner, 2012). However, a pure NH_4_^+^ nutrition has been shown to cause the development of toxicity symptoms in many herbaceous plants, as well as to inhibit NO_3_^−^ uptake (Kronzucker et al., 1999). Therefore, a balanced N diet (NO_3_^−^ and NH_4_^+^) is clearly beneficial for several plant species as compared to that based exclusively on NO_3_^−^ (Forde and Clarkson, 1999). Accordingly, it has been observed in tomato plants that the root growth was optimal when NO_3_^−^ and NH_4_^+^ were supplied in a 3:1 ratio; on the contrary, when NH_4_^+^ concentration was too high, a strong inhibition in the root development was observed (Bloom et al., 1993). A more recent study in watermelon plants showed that with decreasing NO_3_^−^/NH_4_^+^ ratio, the leaf number, leaf area, shoot height, net photosynthesis, biomass, and root growth significantly decreased, as well as the concentration of several macronutrients (Na et al., 2014). Nevertheless, it has also been observed that the optimal NO_3_^−^/ NH_4_^+^ ratio to be applied for cultivation can be dependent on both the plant species and the environmental conditions (e.g., salinity) (Errebhi and Wilcox, 1990). For instance, in canola plants grown in salinity conditions, a NO_3_^−^/NH_4_^+^ ratio of 1:1 is suitable to minimize the negative effects produced by the abiotic stress, thus maximizing the yield and quality (Bybordi et al., 2012). Overall, these results clearly indicate that not only the forms of N but also the concurrently presence of more than one form and in specific ratios among these forms are decisive for specific productive objectives.

Also in the case of Fe nutrition, the nature/forms of the Fe sources utilized can play an important role not only at the level of its uptake but also in the Fe allocation within the plant. In natural environments (soil grown cultures), Fe is present in inorganic mineral forms (e.g., goethite, hematite, and ferrihydrite), which are poorly bioavailable (Colombo et al., 2013; Mimmo et al., 2014). Therefore, plants need to rely on a series of natural ligands for the mobilization of the micronutrient from the insoluble sources and to guarantee appropriate levels of Fe availability (Cesco et al., 2000; Tomasi et al., 2013). However, often this pool of Fe sources cannot satisfactorily sustain an equilibrate growth of plants triggering the onset of Fe deficiency symptoms. The Fe supply (in terms of quantities and usable forms) is an issue also for soilless crops. Several pieces of research have compared in hydroponic solutions the efficiency of different organic ligands complexed with Fe as micronutrient source for plants (Pinton et al., 1999; Tomasi et al., 2009, 2013, 2014). These reports clearly demonstrated that Fe complexed by water-extractable humic substance (WEHS) fractions can be used with higher efficiency than other natural or synthetic sources (Pinton et al., 1999; Tomasi et al., 2009; Zanin et al., 2019), favoring a faster recovery from the deficiency symptoms (Tomasi et al., 2014). The effect appears to be not exclusively ascribable to phenomena occurring at the root level (Tomasi et al., 2013; Zamboni et al., 2016), but also to those happening in the shoot/leaves (Tomasi et al., 2009, 2014). Therefore, from these experimental evidences, it is clear that different Fe sources can be used with different efficiencies, which are the result of a complex response, including biochemical-physiological-molecular aspects, involving the whole plant. However, despite all this knowledge, little still exists in the literature in relation to the application of these findings in the soilless cultures at the production scale. This aspect is even more critical if the increasing demand for Fe-enriched vegetable products is considered.

When nutrient ions are analyzed in hydroponic solutions, one aspect to consider is also the nature of the counter-ion forming the ion pair in solution. In fact, it is well demonstrated that anion which is taken up relatively slowly can also reduce the uptake speed of its counter-ion, as observed for SO_4_^2−^ on potassium (K^+^) uptake (Marschner, 2012). Also, the root capability to extrude protons in the external medium, generating the electrochemical gradient across the plasma membrane at the base of the active nutrient uptake, is consistently influenced by this phenomenon (Pinton et al., 1997). With respect to the hydroponic solutions of soilless cultures, the simultaneous presence of different cations and anions (also at different concentrations) unfortunately makes the phenomenon even more complex and difficult to understand and to manage. However, although the impact of this phenomenon on the overall process of radical nutrient acquisition has long been clear, up to now, there are no specific studies applied to the hydroponic system.

### Nutrient Interactions

It is well known that, at the level of the nutrient acquisition mechanisms of roots, competitive or antagonistic phenomena among elements can occur (Marschner, 2012), leading, in some contexts, also to relevant nutritional disorders affecting the whole plant. A clear example is represented by the interaction between NH_4_^+^ and K^+^. The acquisition of NH_4_^+^ involves an active transport which is very specific for the cation (von Wirén and Merrick, 2004). Differently, K^+^ acquisition is described as a biphasic transmembrane transport of high and low affinity transporters (Gupta et al., 2008; Zhang et al., 2012; Shin, 2014). However, the selectivity of these K^+^ transporters is definitely lower than that of others, including the NH_4_^+^ ones (Gierth and Mäser, 2007). In this regard, it is interesting to note that an inhibition of K^+^ uptake by NH_4_^+^ has been already described, whereas the vice versa does not happen (Mengel et al., 1976; Rufty et al., 1982; Shaviv et al., 1987). This phenomenon has been ascribed to the capability of K^+^ transporters to catalyze also the transmembrane transport also of NH_4_^+^ (ten Hoopen et al., 2010). Certainly, the similarity of the two cations (NH_4_^+^ and K^+^) in terms of valence and ion diameter is, at least in part, at the base of the phenomenon. From the agronomical point of view, this aspect could be crucial for NH_4_^+^-fed plants when exposed to a sub-optimal/unbalanced availability of K^+^ because the competition could induce/exacerbate K^+^ deficiency (Zhang et al., 2010). This aspect could be of particular relevance when the additional application of NH_4_^+^ is of pivotal role to achieve specific qualitative objectives of the edible fruits (Valentinuzzi et al., 2018).

It is also worth mentioning that the interactions K^+^/Na^+^ and Cl^−^/NO_3_^−^ could represent a limiting factor for soilless cultivation of crop plants, especially in semiarid environment characterized by saline water. It is well known that NaCl interferes with the uptake processes of both K^+^ and NO_3_^−^, since K^+^ is sensitive to sodium (Na^+^) in the external environment, while the uptake of NO_3_^−^ is inhibited by chloride (Cl^−^) (Silberbush and Ben-Asher, 1989). This phenomenon could be even more pronounced in hydroponic solutions particularly when used for more than one cycle (closed system) (i.e., exhausted hydroponic solutions).

A further example of competitive interaction between nutrients is that occurring among Ca^2+^, Mg^2+^, and K^+^ (Marschner, 2012), although observed up to now only in soil-grown crops. It is well known that a high availability of K^+^ and Ca^2+^, most often after an unbalanced fertilization practice, can induce Mg^2+^ deficiency in crop plants. At the moment, the mechanisms underlying such interactions have not been clarified. However, as suggested by Schimansky (1981), the excessive availability of the other two cations (K^+^ and Ca^2+^) could reasonably inhibit Mg^2+^ uptake by roots. In this context, and in particular with respect to hydroponic cultures, monitoring the ratio between these three cations in the solutions is without any doubt advisable in order to avoid K^+^/Ca^2+^-induced Mg^2+^ deficiency, particularly when closed systems are considered.

Also Fe acquisition mechanism offers the possibility to describe several examples of interactions among elements/nutrients. In dicots and non-graminaceous monocots, Fe is taken up through the IRT1 transporter (Connolly et al., 2003), which was shown to transport also other divalent cations such as Mn^2+^, Zn^2+^, and Cu^2+^ (Korshunova et al., 1999). In sub-optimal availability of Fe (Zamboni et al., 2016), a marked accumulation of these elements in plant tissue has been observed, with also a different spatial allocation of each element (Tomasi et al., 2014). On the other hand, a rather worrying aspect affecting the quality of the edible plant tissue is that the IRT1 transporter can also mediate the uptake of toxic elements like cadmium (Cd) (Astolfi et al., 2012, 2014; He et al., 2017). This last evidence clearly highlights how crucial is the quality of the nutrient sources adopted as well as that of the water used to prepare the hydroponic solution (in terms of trace elements), to avoid unexpected contamination of crops, the worsening of the edible parts’ quality, and threatening the food safety (Zhao et al., 2010).

Another interesting example of nutrient interaction has been described by Prosser et al. (2001) using S-starved spinach plants (Prosser et al., 2001). In this experience, the authors recorded as a consequence of S starvation a consistent accumulation of NO_3_^−^ in leaves of plants, one of the greatest issues concerning hydroponic productions (Santamaria, 2006). It is interesting to note that this phenomenon seems to be not specific for S, since it has also been observed for Fe-starved cucumber plants (Nikolic et al., 2007). In both these cases, although the uptake of NO_3_^−^ was hampered by the two nutrient shortages (S or Fe), the effect on the assimilation process seems to play a dominant role in determining the NO_3_^−^ accumulation at the leaf level. However, such nutrient interactions (S vs. N and Fe vs. N) seem to be pretty complex and not so easy to simply explain in terms of competition/antagonism phenomena for their transmembrane transporters. In fact, it has also been demonstrated that Fe uptake and the mechanism underlying the process (i.e., the Fe^3+^ reduction step mediated by the plasma membrane reductase FRO) are strongly influenced by the availability of N (Nikolic et al., 2007). Interestingly, Zuchi et al. (2009) have demonstrated that the same Fe acquisition mechanism is severely affected also by S availability. Although the overall impact in terms of nutrient acquisition appears to be essentially the same, the components of the transport machinery seem to be differentially affected by the starvation of the two nutrients; in fact, while FRO is inhibited by both N and S starvation, the transport of Fe^2+^ through IRT1 seems to be hindered only by limited availability of S. These data highlight that the uptake of each single element (e.g., N, S, or Fe) is not exclusively dependent on its availability in the hydroponic solution but also on the presence (and availability levels) of other elements. Thus, it appears evident that an optimized and well-balanced supply of nutrients is a prerequisite for an efficient use of the resources by hydroponically grown vegetables, not only to ensure a high yield but also to guarantee the quality of the edible tissues. Interestingly, it has been demonstrated that a higher Fe accumulation in plant tissues (Fe fortification) can be reached by means of a sulfate over-fertilization (Zuchi et al., 2012).

In the context of nutrient interactions, it is interesting to highlight that these phenomena have been observed also in the case of biofortification programs like with Se. Generally, the biofortification of vegetables is obtained by the application of Se, in the form of selenate, in the nutrient solution, this form being the most available for plants (Schiavon and Pilon-Smits, 2017). Due to the similarities in the chemical and physical features (Shibagaki et al., 2002; El Kassis et al., 2007), the acquisition of selenate follows the same pathway exploited by SO_4_^2−^, based on the sulfate transporters SULTR (Malagoli et al., 2015). For this reason, it is clear that, competing for the same transporter, a sort of antagonism between sulfate and selenate can occur. As demonstrated by (Hopper and Parker, 1999), a high availability of sulfate might prevent the acquisition of selenate, thus vanishing the potential biofortification of the edible parts.

Differently, the experiences aimed at Si biofortification in agricultural crops, both ready to eat and to be transformed (Gottardi et al., 2012; Montesano et al., 2016; D’Imperio et al., 2016a), did not reveal interaction phenomena with other nutrients, at least with negative outcome on the plants’ whole nutrient balance. Several authors have reported a mitigation effect of Si in P-starved crop plants in soilless cultivation systems (Ma and Takahashi, 1989; Ma, 2004); similarly, it has been observed that the supplementation of Si in the nutrient solution of Fe-deficient dicots can alleviate Fe chlorosis (Gonzalo et al., 2013; Pavlovic et al., 2013). Indeed, Gottardi et al. (2012) showed that the Fe reduction and uptake rate at root level were up-regulated by Si supplementation, although Fe translocation to leaves was not influenced. On the other hand, the effect of Si on Fe uptake/allocation in dicots is still debated (Liang et al., 2015). In addition, the Si fertilization might not be only important for Si biofortification approaches, but also to improve plant fitness. The application of silicate fertilizers to paddy fields worldwide has resulted in an increased yield in rice production, with a higher photosynthesis rate and a better light interception due to an improved leaf blade position (Tamai and Ma, 2008). These last aspects, even if observed in soil-grown crops, should also be taken into consideration for soilless cultures for the undoubted advantages that they could guarantee.

## Perspectives

The issues described earlier clearly highlight how unexpected physicochemical phenomena happening in the hydroponic solution can easily modify its composition as well as a series of nutrient interactions can seriously alter the efficiency of the nutrient acquisition process of crops. These phenomena considered together are able, unfortunately, to considerably affect the production of the soilless system based on hydroponics, both from a quantitative and qualitative point of view. For this reason, the availability of new forms of nutrients (nanoparticles, section “Nanoparticles”) and/or of bioeffectors able to enhance the functionality of the root nutrient acquisition mechanisms (PGPRs, section “Use of Plant Growth-Promoting Rhizobacteria in Hydroponic Solutions”) may be of particular relevance. Similarly, the possibility of exploiting tools to monitor the composition of a nutrient solution in *realtime* (sensors, section “*Realtime* Monitoring of Hydroponic Solutions *via* Sensors”) and to analyze the data (interpretation algorithms, section “Interpretation Algorithms and Smart Agriculture”) for a prompt correction may clearly facilitate a more efficient use of the hydroponic solutions.

### Nanoparticles

In the agricultural context, the use of nanoparticles (NPs) is mainly aimed at reducing nutrient losses in the environment as well as at increasing yields through an optimal management of nutrients and water. In fact, thanks to their high specific surface and relevant reactivity (having a particle size lower than 100 nm), nanoparticles might supply the plant with more soluble and available forms of nutrients limiting precipitation and insolubilization processes often described for several fertilizers (e.g., phosphate ones) (Liu and Lal, 2015). For this reason, in comparison to the traditional fertilizers, nanoparticles are considered much more efficient carriers of nutrients for plants (Nair et al., 2010; Campos et al., 2014; Roosta et al., 2017). These advantageous aspects are valid not only for the soil system but even more for the soilless systems (considering also what described for chemical equilibria in hydroponic solution, section “Chemical Management of Nutrient Availability in the Hydroponic Solution”). Therefore, nanoparticles surely represent a promising tool in general, specifically for the soilless growing systems. The use of nanoparticles in soilless cultivation has also been evaluated in the ability to control potential pathogens during cultivation through the use of NP of silver (Ag), Cu, Si, titanium (Ti), and Zn improving the plant defense (Amooaghaie, 2011). Elmer and White (2016) tested the use of metallic oxide nanoparticle to enhance tomatoes’ and eggplants’ growth. Their results showed that NP of CuO increased fresh weights by 64%, reducing the *Verticillium wilt* fungus by 69%, having 32% more Cu in the roots. Within metallic NP, growth enhancement was recorded also for hydroponic spinach treated with iron oxide (Fe_2_O_3_) NP (Jeyasubramanian et al., 2016). The mechanism of Fe uptake by the spinach plant from the Fe_2_O_3_ NP can be explained as follows: in general, the uptake of Fe^3+^ is found to be pH sensitive and naturally Fe^3+^ is insoluble which will be slowly converted into Fe^2+^ under acidic environment. This determined an increase in root and shoot length, biomass, and Fe content in a dose-dependent manner. NP can also be used to recover waste nutrient solution from soilless cultivation systems. An example is given by the photocatalytic treatment of waste nutrient solution coming from tomato cultivation in rice hull substrate (Miyama et al., 2009). In this experiment, the substrate was treated with TiO_2_-coated porous alumina filter, that when irradiated with ultraviolet light, exhibits a strong oxidation effect, decomposing organic compounds. By this process, the phytotoxic compounds could be decomposed and detoxified and the nutrient solution recycled. Moreover, it was observed that tomato growth in the photocatalytically treated system was significantly higher than control in six experiments over 3 years and yields were comparable to those in a currently used open cultivation system using stonewool substrate. In addition to these interesting aspects, it has been clearly ascertained that nanoparticles are able also to affect key processes of plants including germination, seedling vigor, root growth, photosynthesis, and even flowering (Lin and Xing, 2007; Tripathi et al., 2016). Moreover, a protective action of nanoparticles against oxidative stress in plants has been recently demonstrated; the phenomenon was ascribed to the capability of these particles to mimic the role played by antioxidant enzymes (e.g., superoxide dismutase, catalase, and peroxidase) (Burman et al., 2013). Khan et al., (2017) have shown that other abiotic stresses (such as temperature, salinity, drought stress) can be alleviated by nanoparticles as well as drought-stress resistance in plants can be enhanced by applying nanoparticles (Tripathi et al., 2017). In particular, the application of NPs of analcite (Zaimenko et al., 2014) significantly alleviated the drought stress in wheat and corn by increasing the photosynthetic pigments and the accumulation of protective antioxidants in these plant species. Recently, nanomaterials are used as an important tool for increasing the growth and yield of crops under salinity condition (Khan et al., 2017). The application of nano-Si for example significantly reduced salt stress, increasing seed germination and antioxidative enzyme activity, photosynthetic rate, and leaf water content (Haghighi and Pessarakli, 2013; Qados, 2015). In addition, these mitigating effects have been also noticed in other plant species such *Ocimum basilicum* (Kalteh et al., 2018) *Cucurbita pepo* (Siddiqui et al., 2014), and *Vicia faba* (Qados and Moftah, 2014).

With respect to temperature stress, this can either be due to too high or too low temperature. Loss of fluidity of membranes and leakage of solutes are typical symptoms of cold stress. In this regard, nanomaterials such as TiO_2_ may help in alleviating the dangerous effects of cold stress by limiting the membrane damages and electrolyte leakage (Mohammadi et al., 2013). On the other hand, heat stress accelerates the overproduction of reactive oxygen species and increases oxidative stress leading to disintegration of membrane lipids, leakage of electrolytes, and denaturation of biomolecules (Karuppanapandian et al., 2011). It has been shown that, among nanomaterials, low concentration of selenium (Se) can alleviate the effects of heat stress for its antioxidative properties (Haghighi et al., 2014).

However, despite these pieces of evidence, there are still several open questions about the molecular mechanisms underlying the aforementioned phenomena. In addition, the phytotoxic effects of nanoparticles (presumably *via* an enhanced production of reactive oxygen species) described by Khan et al. (2017) make the issue even more complex and less clear. Moreover, little is yet known about their capacity to enter, through the edible tissues, in the food chain and, even more critical, about what effects they may have on human health and the environment. For these reasons, right now, the exploitation at the field scale of these particles appears to be quite premature; in fact, a cautionary principle reasonably prevails. In summary, despite nanoparticles representing a promising tool, further studies are needed to evaluate the impacts on crop growth, quality, and safety (Gardea-Torresdey et al., 2014) before their massive use in the agricultural production system.

### Use of Plant Growth-Promoting Rhizobacteria in Hydroponic Solutions

As widely addressed in literature, the molecular machinery used by plants for the acquisition of mineral elements is characterized by an extreme plasticity in order to guarantee the adaptability to the nutrient fluctuations in the growth medium. Such variations, particularly in the bioavailable nutrient fraction, can be ascribed to several factors, as for instance the concentration and type of nutrient source, the pH, and the redox potential (Tomasi et al., 2009; Marschner, 2012; Mimmo et al., 2014). In addition, a recent body of evidence has drawn the attention to the role played by the plant growth-promoting rhizobacteria (PGPRs) in contributing to the mineral nutrition of plants (Pii et al., 2015). The different mechanisms brought about by PGPRs aimed at increasing the bioavailability of mineral nutrients in the rhizosphere (i.e., atmospheric N_2_ fixation, P solubilization, siderophores production for Fe^3+^ chelation) have been extensively investigated and reviewed (Lugtenberg and Kamilova, 2009; Glick, 2012; Pii et al., 2015, 2019; Alegria Terrazas et al., 2016). Nevertheless, recent pieces of research have highlighted that the PGPRs can themselves induce alteration in the functionality of the molecular machinery devoted to nutrient acquisition (Pii et al., 2015). In this sense, PGPRs were shown to alter the release of protons from wheat roots and other model plants, thus supporting the hypothesis that they could have a direct effect on plasma membrane (PM) H^+^-ATPases (Bashan et al., 1989; Bashan, 1990; Bertrand et al., 2000; Canellas et al., 2002, 2013). Considering that the H^+^ electrochemical gradient at level of the PM is necessary for the absorption of several mineral nutrients, like H_2_PO_4_^−^, SO_4_^2−^, and NO_3_^−^ (White, 2003), the increased PGPR-induced H^+^ release could indeed be reflected in a higher ability of plants to take up nutrients. Similarly, the bacteria *Achromobacter* were shown to induce an increase in the concentration of NO_3_^−^ in plant tissues, most likely for its action on the constitutive high-affinity transport system (cHATS) for NO_3_^−^ (Bertrand et al., 2000; Nacry et al., 2013). Furthermore, the influence of PGPRs was also demonstrated on the molecular mechanisms underpinning the acquisition of Fe (Fe^3+^ reduction – FRO, Fe^2+^ transport – IRT1, and rhizosphere acidification – PM H^+^-ATPase) in dicots plants. The fungus *Trichoderma asperellum* could stimulate Fe uptake in Fe-sufficient cucumber and *Lupinus albus* by enhancing the activity of the root Fe-chelate reductase (de Santiago et al., 2013; Zhao et al., 2014), while *Bacillus subtilis* GB03 could induce the expression of genes coding Fe-chelate reductase and the PM H^+^-ATPase in *Arabidopsis thaliana* (Zhang et al., 2009). Recently, Pii et al. (2016) demonstrated that the PGPR *Azospirillum brasilense* can affect the Fe acquisition machinery in cucumber plants independently from the Fe nutritional status, thus suggesting that the different actors (Fe-chelate reductase, Fe^2+^ transporter, PM H + -ATPase) of the mechanism underlying Fe acquisition in roots can undergo a different regulation following PGPR inoculation. The majority of these studies have been carried out in soil conditions, while very little is known about the real performance of PGPRs in hydroponic systems, where their actions also depend on the ability of thriving and proliferating in specific environments, as well as, on colonizing the plant roots (Lee and Lee, 2015). However, several bacterial strains have been already successfully tested in hydroponically grown fruits and vegetables, obtaining positive effects on the yield and the quality of the agricultural products. The PGPRs belonging to *Bacillus* spp. have been tested for their effect on the growth and productivity of both tomato and pepper plants cultivated in hydroponic conditions (García et al., 2004; Gül et al., 2008). In particular, *B. licheniformis* was shown to significantly enhance the height of plants and the leaf area in both pepper and tomato plants, the effect being species-dependent (García et al., 2004). Nevertheless, the inoculation induced an increase in the number and in the diameter of tomato fruits (García et al., 2004). Similarly, the inoculation of tomato plants with the commercially available PGPR *B. amyloliquefaciens* (FZB24 and FZB42) resulted in an increase in the fruit yield of about 8–9% (Gül et al., 2008). Further studies have demonstrated that the application of specific PGPRs, such as *B. sphaericus* UPMB10 and *A. brasilense* Sp7, which are able to carry out the biological nitrogen (N_2_) fixation, might contribute to reducing the external input of nitrogen sources in the hydroponic solution used for the soilless cultivation of banana plants, still guaranteeing an adequate plant production (Baset Mia et al., 2010). Interestingly, *A. brasilense* was also demonstrated to increase the size of strawberries delivered by inoculated plants, to enhance the nutraceutical qualities (i.e., flavonoids, flavonols, and micronutrients concentration) as well as the sweetness index of fruits (Pii et al., 2018). Nonetheless, hydroponically grown plants in indoor systems might be threatened by pathogen attack; therefore, disinfection practices, either physical (e.g., ultraviolet light, gamma radiations) or chemical (e.g., the use of using carbendazim, hymexazol, imidazole, prochloraz triazole), could be required (Lee and Lee, 2015). Indeed, these control methods can result also in a decrease of PGPR population in the hydroponic systems, thus representing a limitation to the application of beneficial microorganisms to the hydroponic cultivation systems (Hibar et al., 2006). On the other hand, the use of PGPRs that feature also biocontrol traits might represent a valuable alternative to the abovementioned disinfection procedures.

Overall, these observations prefigure a very interesting scenario, in which the potential application of beneficial microorganisms (PGPRs) in the hydroponic cultivation of plants could lead to a further improvement in the productivity and in the nutraceutical properties of crops (Lee and Lee, 2015). Indeed, this practice would also impact on the sustainability of the agricultural systems, allowing a rationalization in the use of water resources and in the external inputs of agrochemicals (e.g., fertilizers and pesticides).

### *Realtime* Monitoring of Hydroponic Solutions *via* Sensors

The hydroponic cultivation of small or medium-size fruiting crops, nowadays gaining ever more importance for the high productivity per area of cultivation (Bradley and Marulanda, 2001), often is based on closed-loop systems on field scale. In this case, the hydroponic solutions are often utilized for more than one single culture cycle; therefore, they need to feature high concentrations of mineral elements in order to guarantee an adequate nutrient supply for plants’ growth in the repeated cycles (Tomasi et al., 2015a). However, the selective removal of nutrients due to plant growth, as well as the evapotranspiration process, could in any case change the concentration of nutrients in the hydroponic solution as well as the accumulation of undesired counter-ions (i.e., Na^+^, Cl^−^), and have also an impact on the electrical conductivity (EC) of the solution itself (Wild et al., 1987; Zekki et al., 2019). Since such alterations in the qualitative and quantitative composition of the substrate solution can adversely impact on crop yield and quality, the need of timely tuning the nutrient solutions is of paramount importance to guarantee an adequate production (Cho et al., 2017). A possible tool for the on-line monitoring of hydroponic solutions can be represented by the use of ion-selective electrodes (ISEs) that are also applied for the assessment of drinking water quality (Melzer et al., 2016). Several authors have already applied this technology to measure the multiple components of a nutrient solution in order to ensure the optimal composition required for plant growth (Chen et al., 2011; Cho et al., 2017). However, at present, many studies have been carried out by measuring one or few plant macronutrients at a time (Bailey et al., 1988; Bamsey et al., 2012; Kim et al., 2013; Rius-Ruiz et al., 2014; Vardar et al., 2015), and this prevented obtaining a complete and realistic picture of the elements’ availability in the nutrient solution. In addition, to the best of our knowledge, selective electrodes for the determination of plant micronutrients, as for instance Fe, Cu, and Zn, have not been developed and/or applied yet. In addition, the use of ISEs as on-line monitor systems may present issues related to the signal drift and the reduction in sensitivity over time, due to a continuous exposure to the nutrient solutions, without appropriate calibration procedures (Gutiérrez et al., 2007, 2008; Bamsey et al., 2012; Kim et al., 2013). The development of a new generation of ISEs through the overcoming of the aforementioned technical limitations (i.e., simultaneous macro and microelements measurements, longer signal stability) might represent a useful tool for the on-line and *realtime* monitoring of nutrient solutions, with the aim of satisfying the nutritional requirements of crop plants for optimal growth.

### Interpretation Algorithms and Smart Agriculture

The sensors and the other new technologies described in section “*Realtime* Monitoring of Hydroponic Solutions *via* Sensors” create massive flows of data that should then be analyzed in order to be adequately exploited. Machine learning algorithms (such as neural networks and genetic algorithms) might be applied for self-calibrating and managing the parameters of hydroponic solutions based on sensors data (Morimoto and Hashimoto, 1996; Suhardiyanto et al., 2009). The constant control of the composition and concentrations could in fact allow the recirculation and reuse of nutrient solutions within closed growing systems, thus reducing the economic costs and minimizing the environmental impact of soilless cultivation systems (Jung et al., 2015). Similarly, advanced Big Data analytics and simulation techniques might allow to forecast the quality and quantity of vegetable or fruit production under various conditions – for instance by creating a *realtime* “digital twin” of the real/physical hydroponic system – and in turn to determine the optimal parameters, such as the composition and concentration of the hydroponic nutrient solution; the temperature, humidity, and CO_2_ levels (in case of greenhouses); and the lighting (in case of greenhouses with artificial light). Finally, Internet of Things and cloud computing might be employed to share the data among different farmers and make the data analysis more efficient and effective.

The use of sensors and data analysis tools is fully included in the concept of precision agriculture (PA) – or smart agriculture (SA) as it has been recently labeled – a new paradigm based on the use of information and communication technologies in the cyber-physical farm management cycle. More formally, PA has been defined as a “management strategy that uses information & communication technologies (ICT) to collect data from multiple sources in view of their later use in decisions concerning production activities” (National Research Council, 1997). Originally, this definition was intended to refer to field processes, being mainly focused on the highly automated site-specific approaches, aiming at overcoming the management limits imposed by the relevant spatial variability in field properties. Later, it was extended to many other types of farming systems, such as livestock, viticulture, and orchards. SA is based on the so-called Knowledge Management approach (also renamed as Knowledge Management 4.0 or KM4.0; Calcante and Mazzetto, 2014; Mazzetto et al., 2017; Meško et al., 2017; Neumann, 2018), which includes the following aspects: (1) ensuring an approach highly oriented to the Internet of Things (IoT) knowledge processes; (2) managing and treating Big Data acquired directly from things (= elements of processes and products) and customers (people acting in the system); (3) sharing information between people or things without any limitation; (4) storing all data and information directly in clouds through Internet of Services (IoS); (5) ensuring that all contents are always available online, also to implement any *realtime* automation process; (6) providing information sharing (C2C, C2M) *via* wireless solutions (hyper-connectivity); (7) fostering of predictive analysis in the main maintenance and control tasks.

Hydroponic systems represent an ideal application context for SA (Dinesh et al., 2018) since they are closer to the industrial context: production processes are carried out in contained and more controllable spaces (greenhouses or tunnels); they are more repeatable (lighting, climatic parameters, and nutrient supply can be controlled); and they can be more easily automated. In the hydroponic practices, there are already some technological examples moving just toward such a direction. Fujitsu, ORIX Corporation, and Masuda Seed launched in 2016 the Iwata Smart Agriculture Project, which aims at collecting a wide set of data concerning the optimal parameters (temperature, humidity, CO_2_ levels, and nutrient concentration of hydroponic solutions) for hydroponic production of various seed varieties and sharing them through Internet. The (Big) Data are collected by Fujitsu in a plant factory located in the city of Iwata (Japan) and consisting of several sensorized and fully controlled greenhouses and then analyzed through the Microsoft’s Azure cloud, also relying on machine learning algorithms. They are then made available to farmers (both professional and amateur farmers) through the IoT and cloud computing platform Akisai Food and Agriculture. Ray (2017) provides an overview of various commercial IoT-based agriculture sensor systems, some of which are suitable for hydroponics and aquaponics farming (e.g., Bitponics, Open garden, and Niwa). Other studies (De Silva and De Silva, 2016; Yolanda et al., 2016; Charumathi et al., 2017) propose instead “new” IoT architectures for hydroponics and aquaponics farming, based on sensors, data loggers, actuators, and software tools (e.g., Arduino).

## Conclusions

This overview of some issues affecting nutrient solutions in soilless cultivation systems clearly highlights the main working topics in which the research world is involved in the field. The huge potential offered by this cultivation approach is indisputable and ranges from productive and qualitative advantages to environmental benefits due to higher efficiency in the use of water and nutritional resources. Currently, there are well-studied and tested research areas whose results are commonly exploited in the soilless cultivation such as the NO_3_^−^ management or the crop quality increase by managing the electric conductivity of the solution. On the other side, besides these positive aspects, there are others, more difficult to manage, related to the interactions among nutrients in their acquisition processes and the nutrient dynamics (bio-geochemical cycles) in the hydroponic solution. These aspects represent a significant discriminant for the soilless management and, in some cases, may limit its diffusion, since the growers must possess specific knowledge and detailed skills to cope with and specifically per each crop species. It is interesting to note that parallel to the research areas dealing with these aspects, there are others newly emerging, technologically advanced but still not widely studied, even if they seem to provide promising tools (nanoparticles, PGPRs) for a more efficient use of this hydroponic-based cultivation approach. Nonetheless, a better knowledge concerning the processes underpinning the acquisition of nutrients and their allocation in the different tissues, also in the presence of these promising tools, is of fundamental importance. Moreover, in a context of smart agriculture strategies application, a consistent and pertinent design of the features of the Information System with the related hardware (sensors) and software (algorithms) components is crucial. Perhaps, borrowing these tools from the industrial environment, where the new paradigm of Industry 4.0 is already applied, could be strategic. Moreover, from the practical point of view, the application of this smart approach in the hydroponic production system will unavoidably require a decoupling of the hardware component management (sensors and data loggers, connections, actuators – in charge to the farmer) from that of all the software components (maintenance of database structures/persistency, interpretation algorithms, controlling data consistency, reporting updates). In fact, the complexity of this latter task requires – at least until when a massive employment of native digitals in the agriculture context is achieved – the presence of a service center specialized in smart agriculture.

Overall, the qualitative management of the crop through the nutrient solution is therefore a concrete strategy, already applicable and characterized by completely new perspectives that will help overcoming the current limits.

## Author Contributions

All the authors have equally contributed to the preparation of the manuscript.

### Conflict of Interest Statement

The authors declare that the research was conducted in the absence of any commercial or financial relationships that could be construed as a potential conflict of interest.
